# On the Connection of Rheumatism with Scarlatina

**Published:** 1854-05

**Authors:** Nathaniel J. Haydon

**Affiliations:** Bovey Tracey, near Newton Abbott


### SELECTIONS.

From the London Lancet.

*On the Connexion of Rheumatism with Scarlatina.* By NATHANIEL J. HAYDON, ESQ., M.R.C.S.E., L.S.A.

I have been much struck with the abstract of a paper lately read by Dr. Willshire before the Medical Society of London, "On some Points in the Pathology of Rheumatism in Children." The part most interesting to myself is that on the connexion of rheumatism with scarlatina. I can hardly coincide with the views of Betz, and declare for the actual identity of scarlatina and rheumatism; but I admit there is a most remarkable analogy between the phenomena, especially the secondary phenomena of these affections. In the year 1850 and 1851 I attended several hundred cases of scarlatina. From May to September (1850), in the village of Hennock (containg with two small contiguous hamlets, about 350 souls,) I attended 135 cases of the disorder. I had the opportunity of seeing every case, save one, of watching every case, and noting their symptoms I had never heard of the views of Betz, or of the supposed identity of the two diseases. My own views on the matter were first directed to the close analogy of the two affections from the following circumstance:—Three children were ill in a family. On one of my visits the mother complained of great fatigue, as the whole care of attending these three children had fallen on her, from an elder daughter having been suddenly attacked with rheumatism. I saw the girl, and she had all the phenomena of acute rheumatism, together with fullness around the eyes, loss of color, and pain in the back; she had had no rash, sore-throat, or other usual sign of scarlatina; she had been well up to the night before; she now had albuminuria. I ordered for her a smart calomel-and-jalap purge, and an ounce and a half of lemon-juice three times a day. In three days she was convalescent, and in three more every trace of albuminuria had subsided. I imagine this case to have been one of scarlatina, without eruption, and the first manifest symptoms to have been that arising from disease of the kidney, producing albuminuria. This state of the kidney was very general during the epidemic. I had under my own care more than forty cases of severe albuminuria during the two years.

Another remarkable analogy between rheumatism and albuminuria will be found in the great tendency of the blood to lose its coloring matter. In a fine boy of ten years old, suffering severely from albuminuria, I took blood from the arm; it was remarkably poor in coloring matter, as also in crassamentum, while the fluid part had more the appearance of milk-whey than anything else. This boy recovered very fast after the bleeding, only requiring an occasional purgative. I had occasion to bleed three others, all strong boys from ten to twelve years old. The same deficiency of coloring matter and crassamentum was found in each, though not so much of the whey-like appearance in the serum. Those three also recovered. I had previously, in the three first cases of albuminuria that presented themselves, used warm baths, leeches to the loins, blisters, and saline purgatives, on the plan so strongly recommended by the late Dr. Miller; they died. Among the three children ill in the first house I have alluded to, two were afflicted with albuminuria. Seeing the good effect of the lemon-juice with the elder sister, I gave it to these children, and it succeeded admirably. I have since given it to more than thirty cases of scarlatina, abuminuria, and in only one has it failed, and this case failed more from general neglect than anything else.

At the village I reside in there were about 1100 persons. In 1851 we had scarlet fever there as an epidemic, when I had fifteen cases of acute inflammatory rheumatism under my care I could only trace the diseases as an open sequela to scarlet fever in one, and this case was very remarkable. A married woman, thirty-two years old, had a slight attack of scarlatina. On the rash disappearing she was seized with acute arthritis—I think the most acute attack I ever saw; there was albuminuria. She recovered. I am too old to trust to specifics, but I have very rarely seen an acute arthritis, or an acute attack of scarlatinal albuminuria, that has not been very speedily relieved by lemon-juice and an active purgative; it was so with this woman.

If the views of Betz are correct, perhaps we may arrive at some solution of the mystery—what is the cause of death, in some cases of scarlatina, when the whole power of life seems to be at once subdued—in short, where the first symptom of disease is death? Now, in the only cases which present death, as it were for the first symptom, we have an entire protrastion of the power of the brain. I have seen this occur twice—*i. e.*, sudden coma, death. In one case, in 1851, during the summer, I was suddenly called to see a child ten years old; it had come in from play, complained of sickness, was much convulsed, shortly became paralytic on the left side, then comatose, and death supervened ten hours from the first attack, the usual rash being thickly spread over its skin before death. The child had never shown disease. I could not obtain a post-mortem examination; but we may expect meningitis to have ensued. Now, what was the cause of the latter? Was it meningeal rheumatism? It is said never to occur. I witnessed three cases of death at Hennock in the early stages of scarlatina, each within the fifth day of the eruption, from acute pleuro-pneumonia, with effusion; yet text-book writers say this state of things never happens, except as a symptom of scarlatina maligna. I have never seen meningitis as one of the sequelæ of the disease. There is one characteristic of scarlatina—viz., the extreme frequency of ulceration, particularly of the posterior nares, tonsils, and fauces which so rapidly supervenes on the first inflammatory symptoms which serve to mark it as different from rheumatism, for rheumatism however acute, is certainly not very prone to that state of things (at least in the country) which ends in ulceration; yet there is a strong analogy between the way the inflammatory action of the tonsils may be in many cases controlled, and the way in which we control rheumatism when it has fixed on some particular part—e. g., by the external appl cation of stimulants. In the inflammatory sore-throat of scarlet fever, I have repeatedly applied a liniment (on hot flannel) of fourteen parts of turpentine, one and a half of spirit of camphor, and one and a-half of tincture of cantharides; it produces the most intense irritation. I have seldom seen it fail of giving immediate relief, and checking the ulcerative process,, and rendering more certain the effect of the local application of the nitrate of silver. We have all seen a mustard plaster or other stimulating application relieve a joint afflicted with rheumatic inflammation. We have seen also, celcbral rheumatism quickly relieved by mustard to to the feet and legs, especially if at the same time we have exhibited an opiate. I know we are told that an opiate would do alone, but I have found it fail more than once, though if not contra-indicated by other sympoms, I have always resorted to it in conjunction with counterstimulants. A friend of my own, a young medical man, while a pupil in London, had scarlatina very slightly, but it was followed by a most acute attack of rheumatie fever, with pericarditis, and there was at the same time great functional disturbance of the brain. He is now well, and in practice.

A young man whom I daily see had scarlet fever slightly, seventeen years since. It was followed by acute arthritis, from which he recovered, but with an anchylosed knee. So closely do I consider scarlatina and rheumatism to be related, that I make it a rule on the decline of the rash, to administer lemon juice; and since I have followed this plan I have not lost one single case from the sequelæ of that disease. To Dr. Owen Rees I feel greatly obliged for having so prominently directed the attention of the profession to the use of lemon juice in certain forms of rheumatism, and I feel very sanguine that it will not fail in many of the affections that are considered as secondary to scarlatina, but which, in fact, I believe will be found to depend on a diseased state of the blood, very analogous to the state of the blood in rheumatism. It appears to me to be a very uncertain point if the state of the kidneys is not reduced as a part and parcel of scarlatinal fever. I rather re gard it as a secondary affection, induced by the diseased condition of the blood.

Bovey Tracey, near Newton Abbott, Feb., 1854,

